# Sliding tethered ligands add topological interactions to the toolbox of ligand–receptor design

**DOI:** 10.1038/ncomms9117

**Published:** 2015-09-09

**Authors:** Martin Bauer, Patrick Kékicheff, Jean Iss, Christophe Fajolles, Thierry Charitat, Jean Daillant, Carlos M. Marques

**Affiliations:** 1 Institut Charles Sadron, Université de Strasbourg, CNRS-UPR 22, 67034 Strasbourg Cedex, France; 2 CEA/ IRAMIS/SIS2M/LIONS, UMR 3299 CEA/CNRS, CEA-Saclay bâtiment 125, 91191 Gif-sur-Yvette Cedex, France

## Abstract

Adhesion in the biological realm is mediated by specific lock-and-key interactions between ligand–receptor pairs. These complementary moieties are ubiquitously anchored to substrates by tethers that control the interaction range and the mobility of the ligands and receptors, thus tuning the kinetics and strength of the binding events. Here we add sliding anchoring to the toolbox of ligand–receptor design by developing a family of tethered ligands for which the spacer can slide at the anchoring point. Our results show that this additional sliding degree of freedom changes the nature of the adhesive contact by extending the spatial range over which binding may sustain a significant force. By introducing sliding tethered ligands with self-regulating length, this work paves the way for the development of versatile and reusable bio-adhesive substrates with potential applications for drug delivery and tissue engineering.

## Abstract (web summary)

Biological adhesion is mediated by specific lock and key interactions between ligand receptor pairs. Here Bauer *et al.* engineer tethered ligands for which spacers can slide at the anchoring point to produce versatile bio-adhesive substrates.

## Graphical abstract

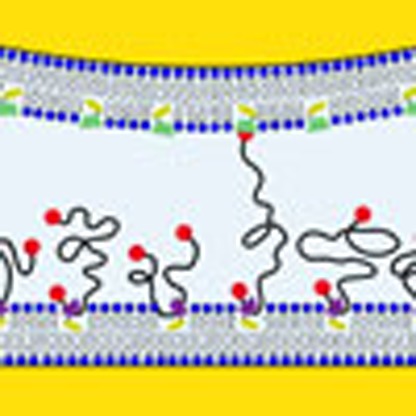

The binding potential for the reaction between a ligand and its complementary receptor extends over a short microscopic length, typically a fraction of a nanometre^1^, as for instance for the typical antigen–antibody bonds^2^. In natural and biomimetic systems, such short reaction ranges would be ineffective in driving adhesion between opposing surfaces, due to the reduced chances of encounter between the corresponding pair moieties. In practice, bio-adhesion requires a spacer, also called a tether, that governs the strength, the kinetics and the range of the binding events. While maintaining the physical attachment of the receptors or the ligands to the substrate, the tether increases the available phase space of their positions and orientations, markedly changing the kinetics and the effective interaction potential of the binding pairs^3^. Often built from a macromolecule, the tether can extend the interaction range between two opposing surfaces up to tens of nanometres, reshaping the surface interaction potential and controlling the final mechanics of adhesion buildup or detachment^4^. The tether is thus the unavoidable keystone of design when one seeks to tailor-make ligand–receptor pairs for specific adhesion^5^.

Optimizing the tether architecture for binders operating between biological substrates under physiological conditions appears as an insuperable challenge. Indeed, the optimal distance from the anchoring surfaces for positioning the complementary ligands and receptors is not, for such substrates, a fixed quantity; it rather depends on time and surface coordinate. Simply choosing a single optimal architecture from the available tether spectrum that includes different lengths, rigidities or chemical moieties cannot in practice solve the design problem since it would require a spatial and temporal optimization of tether parameters^6^. We propose here to tackle this challenge, by introducing a family of spacers with self-adjustable contour length that we coined sliding tethered ligands (STLs). On the basis of topological complexes between polyethylene glycol (PEG) and cholesteryl α-cyclodextrins (CDs), which can be inserted into phospholipid membranes due to their cholesteryl anchor^7^,^8^, STLs combine a ring-shaped anchor through which a polymer chain can slide, while polymer escaping from the ring is prevented by end-attached ligands. Theoretical work on these sliding polymer tethers suggests that they are able to adapt their conformation to external conditions^9^, the sliding character of the topological complex formed by the polymer and the ring is expected to translate into more effective binding and smoother adhesive detachment.

## Results

**Figure 1:**
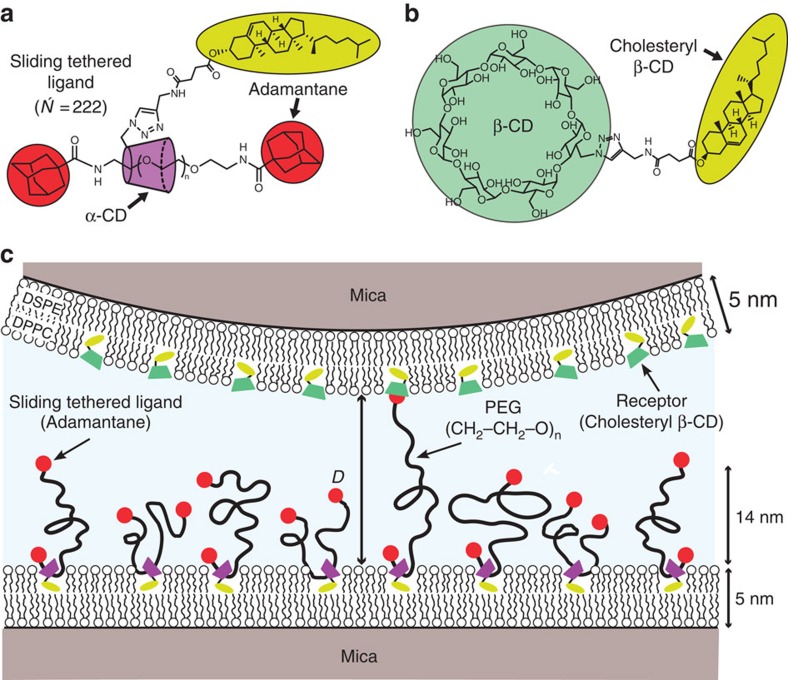
Experimental geometry. (**a**) Molecular structure of a STL. (**b**) The cholesteryl β-CD receptor. (**c**) Molecular configuration used in the SFA experiments. Both membranes deposited on mica by a Langmuir–Blodgett technique were in a gel phase so as to minimize lateral mobility. The surface number density of STLs was *σ=*0.044 nm^−2^, which corresponds to 23 nm^2^ for each tethered ligand molecule (94:6 DPPC:STL). The STLs formed a polymer brush of mean thickness *h*_*0*_=14 nm, as determined from SFA compression curves and confirmed by neutron reflectivity experiments. Each STL is composed of a PEG polymer (*N* =222), which is threaded through the cavity of a cholesteryl α-CD membrane anchor and capped with adamantane at each end. There are on average 1.8 cholesteryl α-CD anchors per chain, as determined by nuclear magnetic resonance. In the opposing surface the ratio DPPC:cholesteryl β-CD is 90:10. The distance between opposing surfaces, *D*, is referred to and measured from the outer edge of the lipid head groups.

The system and experimental setup of this work is shown in Fig. 1. STLs were synthesized (Supplementary Fig. 1) with a PEG tether (*N*=222) threaded through the cavity of a cholesteryl α-CD and end-capped with adamantane at both chain ends (Fig. 1a). Since adamantane forms a host–guest complex with β-CD^10^,^11^, we also synthesized a cholesteryl β-CD (Fig. 1b and Supplementary Fig. 2), as the receptor. Both the STLs and the cholesteryl β-CDs, at equal surface densities, are inserted into opposing phospholipid membranes (Fig. 1c) by their cholesteryl moieties. The synthesis of all compounds is described in the Supplementary Methods.

### Controlling surface structure of STLs and cholesteryl β-CD

**Figure 2:**
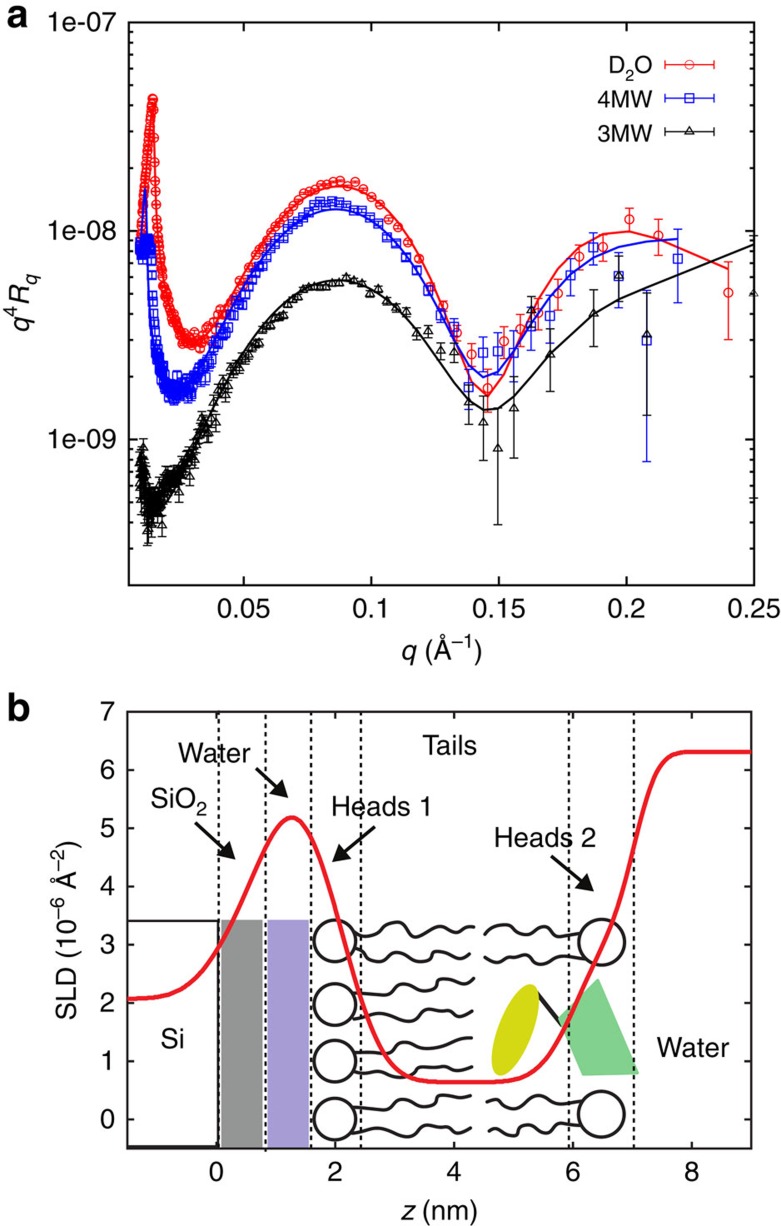
Structure of bilayer with anchored receptors. (**a**) Neutron reflectivity curves for supported bilayer on silicon consisting of a first monolayer DSPE and a second DPPC monolayer with inserted cholesteryl β-CD at 25 °C in water recorded for three different subphase contrasts. (**b**) Corresponding scattering density layer profile obtained with a five-layer fitting model as indicated schematically.

**Figure 3:**
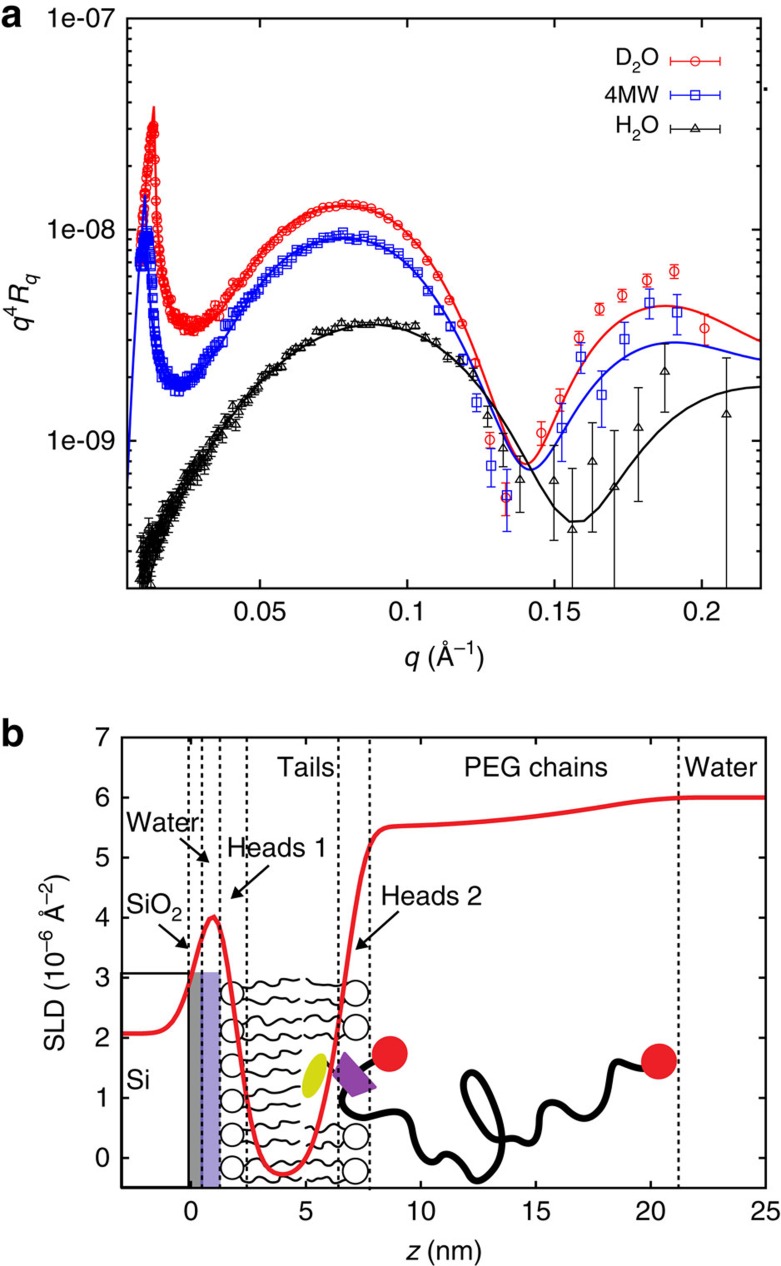
Structure of bilayer with anchored STLs. (**a**) Neutron reflectivity curves for supported bilayer on silicon consisting of a first monolayer DSPE and a second DPPC monolayer with inserted STL at 25 °C in water recorded for three different subphase contrasts. (**b**) Corresponding scattering density layer profile obtained with a six-layer fitting model as indicated schematically.

We studied bilayers modified with STL, as well as cholesteryl β-CD in water with the exact same molecular configuration and with the same chemicals used for the force experiments described below. Figures 2a and 3a display the reflectivity curves for both bilayer samples obtained for three different contrasts and the corresponding best coupled fits. The resulting scattering length density profiles with the schematic structure of the bilayers are shown in Figs 2b and 3b. The fitted parameters are listed in Supplementary Table 4. The DSPE headgroup layer close to the silicon substrate is in good agreement with data already reported in literature^2^,^12^. We found a hydrophobic tail layer thickness of 3.9 nm for the bilayer modified with STL and slightly smaller thickness for the cholesteryl β-CD layer. The measured thicknesses and scattering length densities (SLDs; −0.3 Å^−2^) are as expected for a hydrophobic core in gel phase composed of DSPE and DPPC tails^13^,^14^. A low water content of ca. 10% had to be added due to holes in the bilayer. The presence of the STL and the cholesteryl β-CD in the outer mixed headgroup region leads to a slightly increased thickness compared with the bare DPPC layer (1 nm compared to 0.9 nm for pure DPPC). For the STL bilayer we obtained a polymer layer with a height *h*=13 nm and a surface density *σ*=0.051 nm^−2^ that are in good agreement with the ones obtained from force experiments as described below.

### Surface force interactions

**Figure 4:**
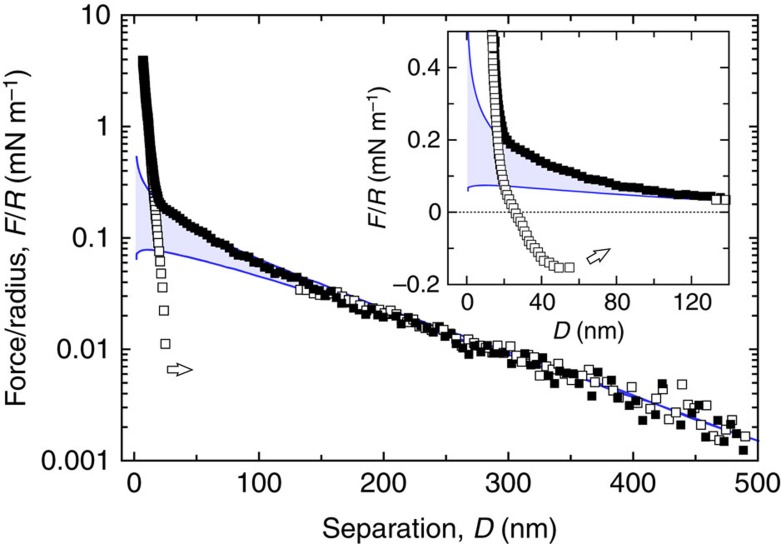
Total surface forces. Measured forces, *F*, normalized by the mean radius of curvature of the surfaces, *R*, between the functionalized bilayers of Fig. 1, as a function of surface separation, *D*. Solid symbols (▪) describe forces on approach and open symbols (□) the corresponding forces on separation. The upper and lower solid lines are fits from a DLVO model^34^,^35^ with, respectively, a constant surface charge *σ*_0_ or a constant surface potential Ψ_0_ boundary conditions (best fit gives *σ*_0_=28 μC m^−2^; Ψ_0_=44 mV). The electrostatic component of the DLVO model is computed from numerical solutions of the nonlinear Poisson–Boltzmann equation^36^ and the attractive van der Waals force is obtained with a calculated non-retarded Hamaker constant *A*_121_=1 × 10^−20^ J using the Lifshitz theory^37^. Note that the DLVO fit is constrained by the long-range exponential nature of the force profile at large separation distances, with a Debye screening length *κ*^−1^=110 nm. At small separations the force–distance profile deviates significantly from the predicted electrostatic repulsion due to the compression of the STL brush. Furthermore, at such strong compressions, the forces acquire an irreversible component and eventually a pull-off force must be applied to wrench the surfaces apart (arrows), as better seen in the linear scale plot of the inset.

Force–distance profiles between the two opposing surfaces displayed in Fig. 1 were measured by the surface force apparatus (SFA) technique^15^ directly after film buildup. As the separation between the surfaces decreases, the forces increase steadily, revealing three distinct regimes in the force–distance profile shown in Fig. 4. Reversible force profiles are observed on compression or separation, as long as the two opposite surfaces do not come into contact (surface separations larger than ∼20 nm). At these large separations, the fully reversible repulsion decays exponentially with a large decay length, *κ*^−1^=110 nm, close to the Debye screening length expected for pure water in equilibrium with dissolved carbonic gas, at pH 5.7. The repulsive forces result from the overlap of the electrical double layers associated with the charged polar heads of the phospholipids. At short approaching distances (<20 nm), the electrical double-layer repulsion is dominated by a steeper repulsion due to the compression of the confined polymer brush. Ultimately, there is a marked change in the repulsive force profile towards a steeper regime at very small separations, which may be ascribed to the steric wall repulsion. Indeed, the primary adhesive minimum observed for bare mica surfaces in air or water can no longer be observed. Comparison with the bare mica contact position leads to a value of 9.8±0.2 nm for the thickness of the two lipid bilayers, a value taken hereafter as the zero reference distance for all force–distance profiles. The forces generated by the sliding tethered ligands can be obtained by subtracting the DLVO (Derjaguin-Landau-Verwey-Overbeek) contribution from the force curves displayed in Fig. 4. They are comprised of a repulsive part due to the compression of the polymer spacers and of an attractive component due to sliding ligand forces as demonstrated below.

### Ruling out nonspecific interactions

**Figure 5:**
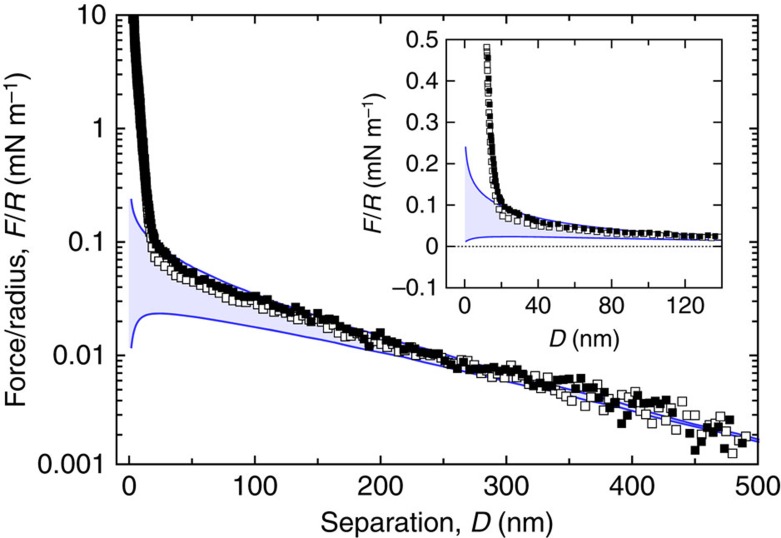
Reference forces between STLs and a surface without receptors. Normalized force–distance profiles between a bilayer modified with STLs and a pure DPPC bilayer on approach (▪) and separation (□) of the surfaces. At large separations the interaction is exponentially repulsive as expected for a double-layer interaction. Solid lines represent the boundary conditions constant surface charge *σ*_0_ (upper curve) and constant surface potential Ψ_0_ (lower curve) calculated as before. The best-fit parameters, Ψ_0_=30 mV and *κ*^−1^=150 nm, are essentially constrained by the long-range exponential repulsive part of the force profile. The inset shows an enlargement of the force–distance profile on a linear scale at short separations. It indicates that the electrical double-layer repulsion operates at almost constant surface charge boundary conditions. At short separations the interaction deviates from the pure electrostatic repulsion due to the compression of the STL chains. When compressing further the surfaces a steric repulsion due to the deposited bilayers on the mica substrates is ultimately encountered. No attractive component is observed when the surfaces are separated (□) even under large applied loads: the observed small hysteresis here reported simply indicates that the local structure has been slightly affected under such conditions.

**Figure 6:**
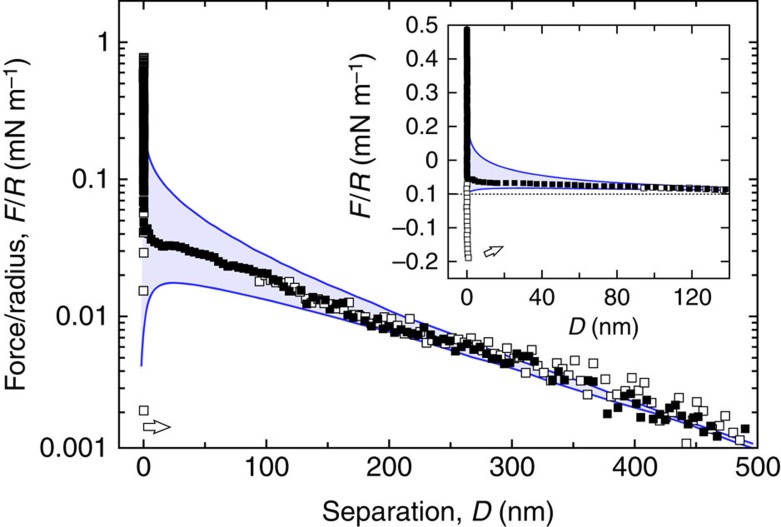
Reference forces between the receptors and a naked bilayer. Normalized force–distance profiles between a bilayer modified with cholesteryl β-CD and a pure DPPC bilayer on approach (▪) and separation (□) of the surfaces. At large separations the interaction is exponentially repulsive as expected for a double-layer interaction. Solid lines are calculated as described before at constant surface charge and constant surface potential boundary conditions with Ψ_0_=25 mV and *κ*^−1^=150 nm. At short separations the repulsion deviates from the pure electrostatics as a steric repulsion due to the presence of the deposited bilayers on mica is encountered. A small pull-off force must be applied to separate the surfaces at contact (arrow): its range and magnitude indicate van der Waals interactions.

To rule out unspecific adhesion, we performed two reference experiments. In both experiments we have used the exact same amounts of STL and cholesteryl β-CD, as for the main experiment. Figure 5 shows the force curves between one DPPC bilayer modified with STL and the opposing bilayer consisting of pure DPPC. They display the steric repulsion due to the polymer compression. As expected^2^,^16^, no adhesive regime was observed for approach as well as withdrawal. Figure 6 displays the reference experiment with one DPPC bilayer modified with the surface density of cholesteryl β-CD. On withdrawal the curves exhibited a very short-ranged adhesion that is due to van der Waals adhesion, which is in good agreement with experiments for pure lipid bilayers previously described in the literature^17^.

### Repulsion from the compressed spacers

**Figure 7:**
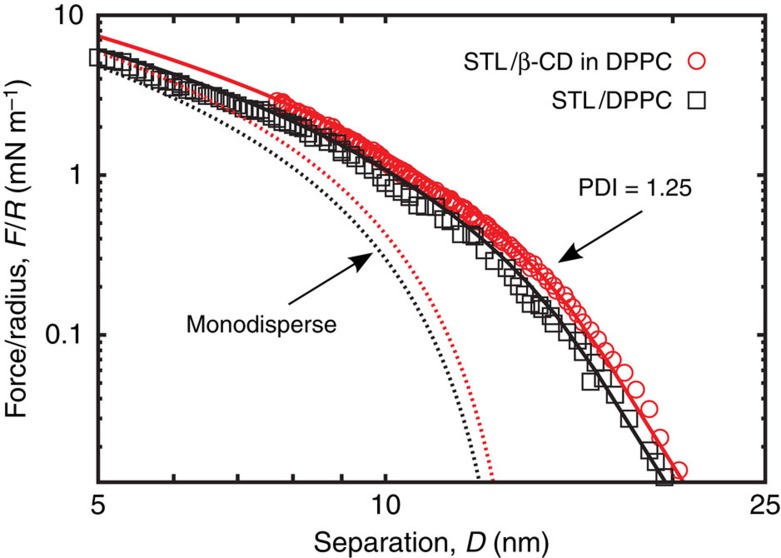
Net repulsive forces induced by STLs on a naked bilayer without receptors. Approach curves after subtraction of the constant charge DLVO contribution, for the sample architecture displayed in Fig. 1 and for the reference experiment with the same STL content but without cholesteryl β-CD receptor. The dashed lines correspond to Milner–Witten–Cates (MWC) fits^18^,^19^,^20^ for monodisperse polymers. The full lines represent MWC fits numerically corrected for polydispersity (polydispersity index=1.25). As expected we find similar brush heights for both samples: *h*_0_=13.8±0.5 nm without β-CD and 14.2±0.5 nm with β-CD.

Figure 7 shows the polymer compression forces under approach, *F*_rep_, extracted from both the adhesive geometry and the non-adhesive case where the top layer does not carry cholesteryl β-CD. We follow the method and notations of Balko *et al.*^18^ that implemented the Milner–Witten–Cates model^19^,^20^ to fit the force profiles under approaching conditions. Experiments and theory (full lines) are in excellent agreement over a large range of compressions, from the onset of the repulsion, where the repulsive forces vanish, up to high compressions at small distances. With the given polydispersity index=1.25, one obtains a brush height *h*_*0*_=14 nm, corresponding to a STL surface number density *σ=*0.044 nm^−2^ using an ethylene glycol monomer size *a*=3.5 Å (ref. 21), which is close to the number density measured by neutron reflectivity^22^ (see also Supplementary Table 2).

### Specific adhesion forces resulting from ligand sliding

**Figure 8:**
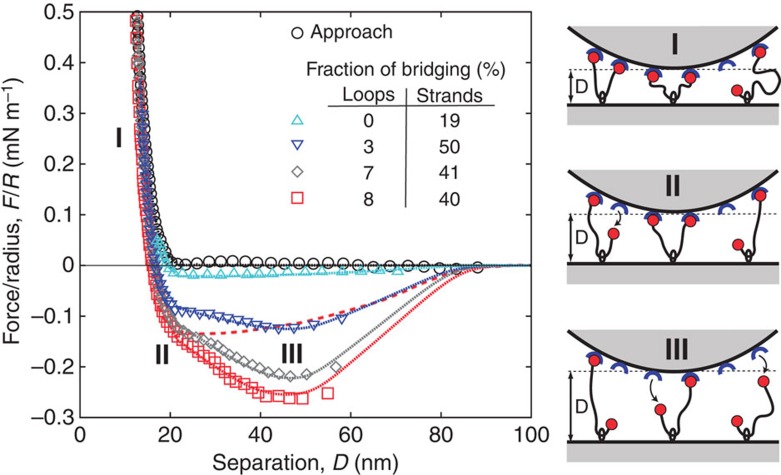
Net attractive forces induced by STLs mediated specific interactions. Force profiles obtained on approach (circles) and on separation (all other symbols) after subtraction of DLVO contribution on the same sample spot successively increasing the compression on approach. The full lines represent fits of the force profiles according to the model described by equation (1) with parameters summarized in Supplementary Table 3. The coarse dashed line represents the expected attraction curves without interconversion, for the same total number of bridges as the red curve (squares). On the right hand one can find a schematic illustration of the proposed mechanism for the interconversion of double-bridging STLs into single-bridging STLs, responsible for the unique force profiles.

The forces under separation are shown in Fig. 8. At short distances the forces are repulsive but become attractive as the separation distance increases. Note that the attractive profile does not present a dependence on the resting time at contact under an applied load (up to a few hours at fixed load) nor on the number of cycles (load/unload) previously performed at the same contact position. Attraction is due to the bridging forces induced by the specific binding between the adamantane end group of the tethers and the β-CD receptor on the opposing surface. Indeed, as shown above, no attractions are observed in the absence of either the functional adamantane chain end or the β-CD groups. The amplitude of the attraction increases as the minimum distance of approach decreases, as one would expect from the evolution of the bridging probability as a function of minimum approach distance, *D*^min^.

## Discussion

**Figure 9:**
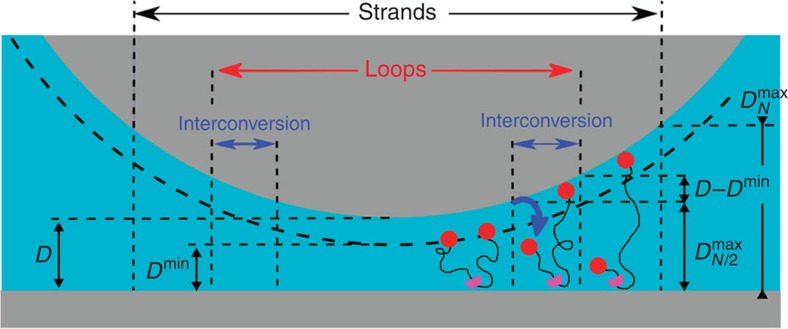
Schematics of bond interconversion. For opposite curved surfaces separated by a distance *D*, bridging loops can exist only in the central region of the gap provided the distance between the anchoring ring and the ending caps is smaller than 
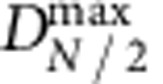
 while bridging single strands may exist up to separations 
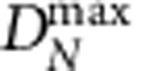
. When the two opposite surfaces are separated from the minimal separation *D*^min^ set after brush compression, one extremity of a bridging loop will detach as soon as the distance between its anchoring ring and one of its extremity becomes larger than 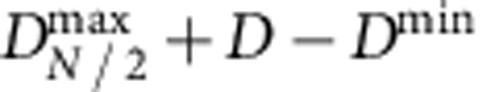; in this namely ‘interconversion zone' this former bridging loop has been transformed into a new single strand. In this schematic all the other STLs of the brush, which are not bridging the two opposite surfaces, have not been represented for clarity.

Attractive forces due to tethered ligand–receptor interactions have been previously observed and quantitatively explained by a combination of polymer and ligand binding theories^2^,^3^,^21^,^23^,^24^. However, the actual force profiles of Fig. 8 present marked differences with the usual U-shaped profiles of such systems^2^,^3^—see Fig. 8 and Supplementary Fig. 3 for the non-sliding profile expected in our case—suggesting a subtle role of the sliding character of our tethers. To quantitatively explain the STL force profiles we write the STL contribution to the total force *F* between the two SFA surfaces of radius of curvature *R* as the sum of the repulsive component measured and analysed beforehand, *F*_rep_, and all attractive contributions from the individual chains as:


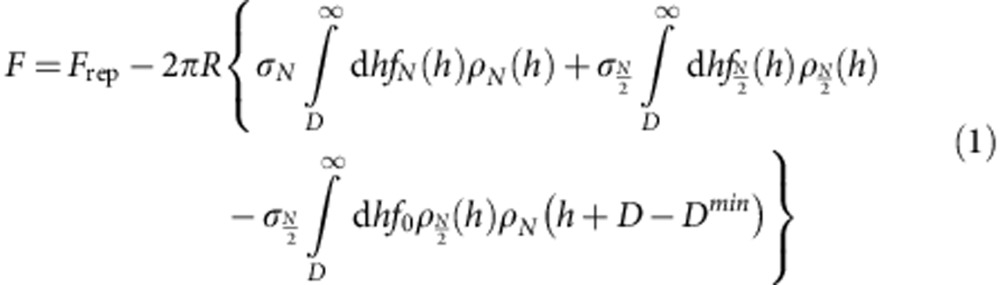


where *σ*_*N*_ and 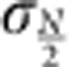 are, respectively, the surface densities of single-end and double-end bridges that we refer to as strands and loops (see right panels in Figs 8 and 9), 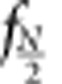 and *f*_*N*_ are, respectively, the attractive forces exerted by the loops and the strands, *f*_0_ is a constant force required to pull the chains through the CD rings, and 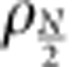 and *ρ*_*N*_ are cutoff functions accounting for the dissociation of the adamantane/β-CD host–guest complex. The three attractive terms of this equation correspond to the three regions in Fig. 9. The first term refers to the attractive forces of single strands, the second term the attractive forces of loops and the third term the forces generated by the interconversion mechanism. For the strands bridging between opposite walls at distance *h*, we consider forces and ranges of attraction described by:


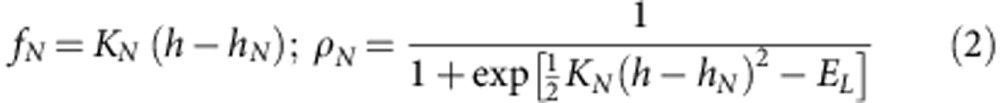


where the forces *f*_*N*_ are in practice given by harmonic spring contribution with a spring constant 
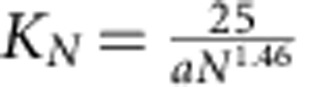
 and an equilibrium position *h*_*N*_=1.05*aN*^0.65^ with *a*=0.35 nm. The values of *K*_*N*_ and *h*_*N*_ were obtained by numerical simulations and shown before to adequately describe stretching of polymer chains with the distance range relevant for our experiments^3^. Given the ligand–receptor band strength *E*_L_ (here *E*_L_=10 in *k*_B_*T* units, calculated from the binding constant 5 × 10^4^ M^−1^ (refs 10, 11, 25)) the function *ρ*_*N*_ measures the thermally activated detachment of strands when the stretching energy 
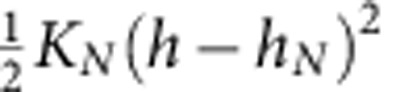
 becomes larger than the bond strength *E*_L_. Forces and range of attraction for bridging loops are described likewise with a monomer number *N*/2 in the equation (2). When one extremity of a bridging loop detaches, the force balance between the two loops halves does not hold any longer, and half of the chain may be pulled through the anchoring CD ring until eventually sliding is prevented by the tether capping moiety. Our fitting shows that the polymer chain does not slide freely through the anchoring ring, but instead a finite force is required to pull the PEO chain through the CD ring. We have modelled this finite force as being constant, *f*_0_, throughout the sliding process. During interconversion, where a freshly detached loop is being stretched until it eventually becomes a strand, its contribution to the total force is described by the third attractive term of equation (1) with *ρ*_*N*_ and 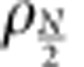 given by equation (2) with *N* and *N*/2, respectively. The product of distributions *ρ*_*N*_ and 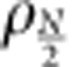 in the third term counts chains freshly detached for distances larger than 
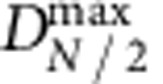
 and not yet reaching the ‘strand state' at distances 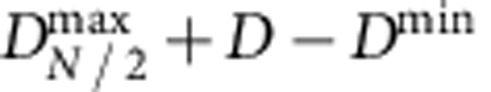.

Fittings of all curves in Fig. 8 were made with a fixed set of parameters, except for the initial relative fraction of bridging loops and strands, indicated in the inset. The quality of the fitting procedure convincingly shows that interconversion between loops and strands, pictured on the right panel of Fig. 8 and assumed for writing equation (1), does indeed control the attractive forces in the STL system. In the interconversion scenario, the initial bonding state of the sliding tethers consists of a mixture of single bridges (the strands), double bridges (the loops) and unbound chains. As the two opposing surfaces are pulled apart, the loops are progressively converted into strands due to the rupture under force of one of the adamantane/β-CD bonds. A freshly ruptured loop is then pulled at constant force *f*_0_ through its CD ring, eventually detaching when the stored stretching energy becomes comparable with the binding energy of ligand–receptor, about 10 *k*_B_*T* units^11^,^25^. Pulling the chains through the rings at constant force is essential to describe the nearly linear decay that precedes the minima of the attractive force profiles in Fig. 3. Such decay is not present in a typical attractive profile for tethered ligand–receptor pairs^2^,^3^, also depicted in Fig. 8. Note that the minima of the measured forces in our case are located at about the same separation indicating that, from the polydisperse composition of chain lengths, chains of *N*=600 contribute most to the withdrawal force profiles. The shapes of attractive profiles measured for STLs are in marked contrast with attractive forces resulting only from chain stretching (Fig. 8 and Supplementary Fig. 3): they provide in practice for a long-range, smoother force profile between the complementary surfaces. To our knowledge this is a new feature for bio-adhesive attractive profiles, observed for the first time here with our STL system.

We have thus designed and built a new family of sliding tethered ligands. The goal of spacer design is to facilitate the formation of the ligand–receptor bond, by attaching the ligand or the receptor to a flexible or semiflexible tether, which enhances the conformational space for ligand orientation^26^ and position^3^. An intrinsic limitation of conventional spacer design with a fixed length is that one typically optimizes spacer length according only to the particular nature of a given adhesion system in drug delivery or tissue engineering. Here, by anchoring the spacer to the substrate with a sliding connection, we effectively provide for a large number of possible spacer lengths within the same molecular architecture. As we have shown, this changes the intimate force profile of the adhesive contact, paving the way for the development of new, more effective and versatile bio-adhesive substrates.

## Methods

### Chemicals

STLs and the cholesteryl β-CD were synthesized in our group, as described in Supplementary Methods, see also Supplementary Table 1 for chemicals used in this work. For STL, we use a PEG tether (*N*=222) threaded through the cavity of a cholesteryl α-CD and end-capped with adamantane at both chain ends. The DPPC (1,2-dipalmitoyl-sn-glycero-3-phosphocholine), DSPE (1,2-distearoyl-sn-glycero-3-phosphoethanolamine) chloroform (stabilized with ethanol) were purchased from Sigma–Aldrich. The ultra-pure water (18.2 MΩ cm) was obtained from a commercial Millipore purification system.

### Surface force apparatus experiments

The solid support consists of thin, molecularly smooth, back-silvered mica sheets, glued onto fused silica hemicylinders with an average radius of curvature *R*≈2 cm. After thickness calibration of the mica sheets, the bilayer samples are prepared by Langmuir–Blodgett deposition. A chloroform–methanol 4:1 solution of lipids, lipid–STL or lipid–CD was spread on the water surface of a Langmuir NIMA trough (10 cm × 30 cm). The isotherms are recorded with a speed of 10 cm^2^ min^−1^ at 25 °C. All samples are prepared with a first layer of DSPE, deposited at 40 mN m^−1^ as a 65:35:8 chloroform:methanol:water solution spread on the water surface. The second layer is always deposited at 30 mN m^−1^ and degased ultra-pure water is used as subphase. The deposition speed is set to 5 mm min^−1^ for the DSPE layer and to 2 mm min^−1^ for the second layer. The transfer ratios have been close to one for the first layer and >0.9 for the second layer.

All force measurements were performed at 25.0 °C with mica surfaces coated with freshly deposited bilayers. The films were studied in pure degased millipore water and kept in aqueous environment at all times to preserve their native structural organization. Force–distance profiles were measured using a home-made device based on the initial version of the Tabor–Israelachvili SFA^15^. As described in detail elsewhere^27^ the instrument allows the force *F* between two mica surfaces (of mean radius of curvature *R*) to be measured to within 10 nN as a function of the determined surface separation *D*, which can be measured to a typical accuracy of 0.2 nm, using multiple beam interferometry^28^. The normalized force *F/R* can be detected to within 0.003 mN m^−1^, while the maximum reliably measurable force will depend on the mechanical compressibility of the entire system. Typically surface deformations occur for applied loads larger than 8–15 mN m^−1^ and *F/R* becomes meaningless due to the deformation of the glue beneath the mica sheet; for that reason, data are only reported for smaller loads, where the measured values of *F/R* correspond to the free energy *E* per unit area^29^. Highly reliable results were obtained by performing measurements under negligible thermal drift between the surfaces, below 0.5 nm min^−1^, it is minutes to the power minus one, at several different contact positions. To maximize the time for achievement of thermodynamic equilibrium, the surface displacements were carried out as slowly as any thermal drift would permit.

### Neutron reflectivity

*Sample preparation*. The bilayers were prepared on 5 × 5 × 1 cm^3^, homogeneously n-doped silicon single crystals, oriented [111] on the side where the film is deposited and atomically smooth with a roughness <5 Å, as determined by the manufacturer (SILTRONIX, Archamps, France). Before each deposition the silicon block was cleaned with chloroform, ethanol and water then treated with ultraviolet/ozone for 30 min to reach a hydrophilicity as high as possible. The double-layer deposition was carried out on a NIMA trough available in the ILL soft matter lab (20 × 30 cm^2^). The first layer of DSPE was deposited at 40 mN m^−1^ by the classical Langmuir–Blodgett technique, whereas the second layer of DPPC, modified either with STL or cholesteryl β-CD, was deposited by the Langmuir–Schaefer method (horizontal sample) at 30 mN m^−1^. The temperature was kept constant at 25 °C. The samples were then inserted into a Teflon sample cell, which was put into an aluminium box to be mounted on the neutron reflectometer and temperature controlled using a water circulation bath. The cell was connected to a solvent circuit by means of a peristaltic pump to be able to change the subphase for different contrast. More detailed information about the substrate and sample preparation has been given elsewhere^13^.

*Instrumental setup*. The measurements were conducted at the D17 reflectometer^30^ operated in time of flight mode at the ILL, Grenoble (France) with a wavelength range from 2 to 20 Å, giving a *q*-range for specular reflectivity of 0.005–0.3 Å^−1^. Each measurement is performed at two reflection angles, *θ*_1_=0.8° (resolution Δ*q*/*q*=2.7%) and *θ*_2_=3.2° (resolution Δ*q*/*q* varied linearly from 3.8 to 13%) (ref. 30). The detector efficiency was calibrated with H_2_O. For the actual experiment the neutron beam enters the silicon substrate through one 5 × 1cm^2^ side of the block, hits at grazing incidence the polished 5 × 5 cm^2^ face on which the layer under study has been deposited, and goes out through the opposite 5 × 1-cm^2^ side^13^. Two direct beams have been measured at the settings of the two angles of incidence for data normalization. Each sample was measured at three different solvent contrasts, such as H_2_O (SLD=−0.56 × 10^−6^ Å^−2^), 4-match water (4MW, SLD=4 × 10^−6^ Å^−2^) and D_2_O (SLD=−6.4 × 10^−6^ Å^−2^) to remove ambiguities of the fits^13^,^31^.

*Data analysis and fitting model*. Specular reflectivity, *R*(*q*), is defined as the ratio between the specularly reflected and incoming intensities of a neutron beam, which is measured as a function of the wave vector transfer, 
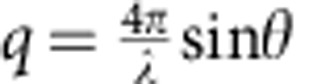
, perpendicular to the reflecting surface, where *θ* is the angle and *λ* the wavelength of the incoming beam. *R*(*q*) is related to the scattering length density profile across the interface by the square modulus of its Fourier transform. Therefore, the phase is lost and the data need to be fitted with an appropriate model to obtain the density profile. In this manner it is possible to determine film profiles within subnanometer precision^13^,^32^. The data are fitted with the ProFit package 6.2 (QuantumSoft), where the specular reflectivity is calculated by the Abeles matrix method for stratified interfaces^33^.

For the cholesteryl β-CD/phospholipid bilayer a five-layer model has been adopted, which has already been used to describe bilayers with amphiphilic CDs^8^, whereas for the STL/phospholipid bilayer sixth layer had to be added to account for polymer^22^. As illustrated by the cartoon in Fig. 2, they both consist of a SiO_2_ layer on the silicon block, a thin water layer, a thin DSPE headgroup slab, a hydrophobic layer composed of phospholipid tails and the cholesteryl residues, as well as an outer DPPC headgroup slab with inserted CD moieties. For the STL bilayer an additional sixth layer with a parabolic density profile was added, widely used to model polymer brushes^2^,^19^,^20^.

The fits for different contrasts have been performed in a coupled manner. Only the subphase scattering length density is changed for different contrasts. The error bars are determined by varying each parameter of the model and evaluating the *χ*^2^ parameter, as well as visually checking the quality of the fit. The results fall within the error bars if they still give satisfactory fits for all measured contrasts. Good coupled fits could be obtained for all measured samples at different temperatures with an exploitable *q*-range from 0.01 to 0.25 Å^−1^. The detailed fitting results can be found in the Supplementary Table 4.

Silicon substrates were first characterized, leading to a SiO_2_ layer, 0.8-nm thick with a roughness of 0.6 nm. These parameters have been constrained to these values for fitting the supported bilayer experiments. For both samples we found a thin water layer with high roughness between substrate and the supported bilayer, which were in good agreement with literature values^13^,^32^.

The surface density of the polymer *σ* was be calculated from the volume density profile with the brush thickness *h* and the polymer volume fraction at the interface Φ_0_, which are both obtained as independent fit parameters from the neutron reflectivity experiments, using with the number of monomers *N* and the molecular volume of an ethylene glycol unit *v*_EG_=0.061 nm^3^ (ref. 12).

## Additional information

**How to cite this article:** Bauer, M. *et al.* Sliding tethered ligands add topological interactions to the toolbox of ligand–receptor design. *Nat. Commun.* 6:8117 doi: 10.1038/ncomms9117 (2015).

## Supplementary Material

Supplementary Information Supplementary Figures 1-3, Supplementary Tables 1-4, Supplementary Methods and Supplementary References
