# Supplementary material for: Sliding tethered ligands add topological interactions to the toolbox of ligand–receptor design
Source: Nat Commun. 2015 Sep 9;6:8117. doi: 10.1038/ncomms9117 (PMC4579401; doi:10.1038/ncomms9117)
Supplement: Supplementary Information — Supplementary Figures 1-3, Supplementary Tables 1-4, Supplementary Methods and Supplementary References [file ncomms9117-s1.pdf]

Supplementary Figures

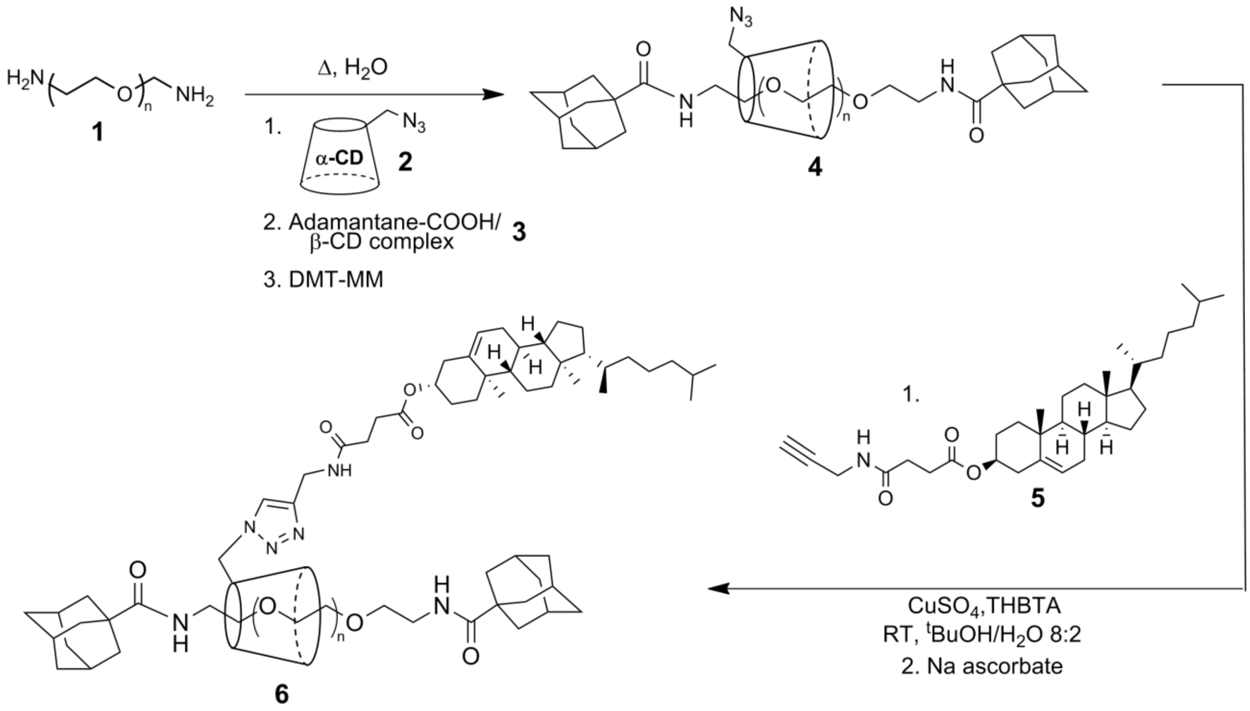

Supplementary Fig. 1. Two-step synthesis of the STL **6**.

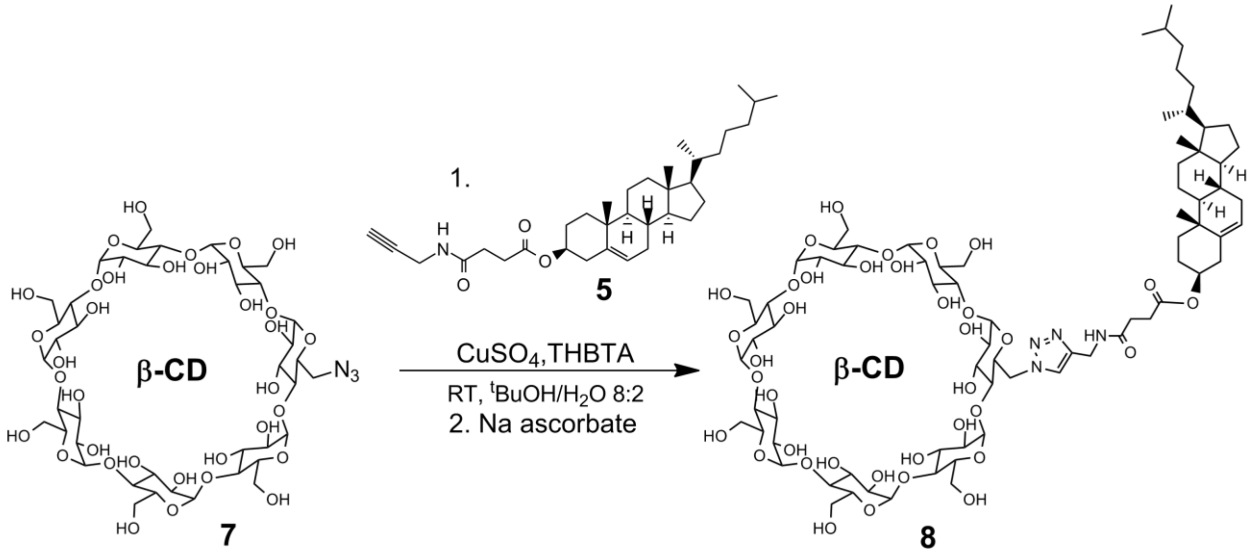

Supplementary Fig. 2. Synthesis of the cholesteryl β-CD receptor **8**.

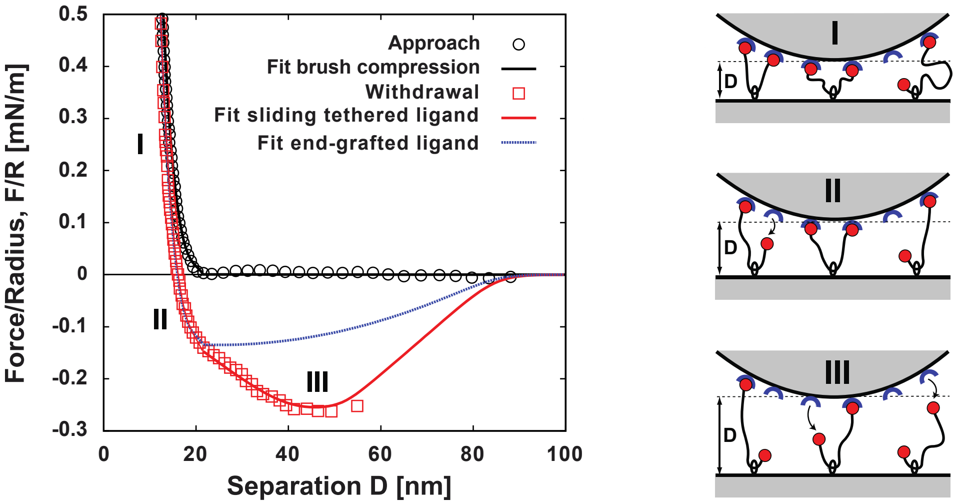

Supplementary Fig. 3. Comparison between two different fits for the pullout experiment with STLs. In blue, best-fit assuming only chain stretching and detachment. In red, the result of fitting with the interconversion mechanism. Note that the almost linear reinforcement of the attractive potential middle range can only be explained by interconversion.

Supplementary Tables

Name	Formula	Molar Mass [g/mol]	Provider
(+)-Sodium-L-ascorbate	$C_6H_7NaO_6$	198.11	Sigma
1-Adamantane carboxylic acid	$C_{11}H_{16}O_2$	180.24	Aldrich
4-Dimethylaminopyridine (DMAP)	$C_7H_{10}N_2$	122.17	Fluka
DMT-MM	$C_{10}H_{17}ClN_4O_3$	276.72	Aldrich
Acetone	CH_3COCH_3	58.08	Sigma-Aldrich
β-CD	$C_{42}H_{70}O_{35}$	1134.98	Wacker
tert-Butanol	$(CH_3)_3COH$	74.12	Fluka
Chloroform	$CHCl_3$	119.38	Sigma-Aldrich
Cholesteryl-hemisuccinate	$C_{31}H_{50}O_4$	486.74	Sigma
Copper(II) sulfate	$CuSO_4$	159.61	Riedel-de Haën
Deuterochloroform	$CDCl_3$	120.38	Eurisotop (France)
Diethylether	$(CH_3CH_2)_2O$	74.12	Sigma-Aldrich
Dimethylsulfoxide-D6 (DMSO-D6)	C_2D_6SO	84.17	Sigma
Methanol	CH_3OH	32.04	Sigma-Aldrich
α-CDN_3	$C_{36}H_{59}N_3O_{29}$	997,86	Biocydex (France)
β-CDN_3	$C_{42}H_{69}N_3O_{34}$	1160,00	own laboratory
bis-amino-PEG-10kD	$H_2N(C_2H_4O)_{222}C_2H_4NH_2$	10000	Aldrich
Propargylamine	$HC_3H_2NH_2$	55.08	Sigma-Aldrich
p-Toluenesulfonic anhydride	$(CH_3C_6H_4SO_2)_2O$	326.38	Sigma-Aldrich

Supplementary Table 1. Chemicals used for the synthesis.

system	h_0 [nm]	p_0h_0 [mN/m]	σ_{brush} [nm^{-2}]	A [nm^2]	$\bar{N}$	PDI
STL/β-CD	14.3	5.0	0.041	22	222	1.25
STL/DPPC	14.0	4.3	0.039	26	222	1.25

Supplementary Table 2. Approach curves displayed in Figure 3 were computed using the MWC model for compression of polymer brushes corrected for polydispersity. The polymer density, σ_{brush}, is calculated from $\sigma_{brush} = (h/a)^3 N^{-3}(\pi^2/12)a^{-2}$ with the size of an ethylene glycol monomer a = 0.35 nm [1]. The available area per polymer chain is $A = \sigma^{-1}$.

system	D^{min} [nm]	h_0 [nm]	N	$\frac{\sigma_N}{\sigma}$ [%]	$\frac{\sigma_{N/2}}{\sigma}$ [%]	f_0 [$k_B T/a$]
STL/β-CD	7.7±0.1	14.3±0.1	640	0.40	0.079±0.001	2.6±0.1
	11.0±0.1	14.3±0.1	630	0.41	0.070±0.001	2.6±0.1
	13.9±0.1	14.3±0.1	630	0.50	0.032±0.001	2.6±0.1
	17.1±0.1	14.3±0.1	630	0.19	0	-

Supplementary Table 3. Results from fitting the withdrawal curves displayed in Figure 8 using equation (1) in the main text. For all fits the binding energy was set to 10 $k_B T$, which is the complexation energy of β-CD and adamantane [2,3]. The distance for maximum compression at each run is denoted by D^{min}, N is the number of monomers of the bridging polymers, $\frac{\sigma_N}{\sigma}$ is the fraction of single bridging polymers (the strands) calculated respectively to the total surface density σ»0.044 nm^{-2} (23 nm^2 per each tethered ligand molecule), and $\frac{\sigma_{N/2}}{\sigma}$ is the double bridging polymer fraction (the loops). Significant variations in the accuracy of the fittings can be seen for deviations of the parameters N, $\boldsymbol{\sigma_N}$ and $\boldsymbol{\sigma_{N/2}}$ larger than a few percent. Note that the low fraction of chains involved in bridging and looping implies that no significant modifications to the repulsive part of the profile can be detected upon separation. Values of the measured friction coefficients correspond to those of a bead of a size of a monomer moving in a liquid ten times more viscous than water.

Layer	Parameters	Cholesteryl β-CD	STL
Water	Thickness [nm]	0.7 ± 0.1	0.5_5 ± 0.1
	SLD [Å^{-2}]	-	-
	Water [v/v%]	100	100
	Roughness [nm]	0.5 ± 0.1	0.6± 0.1
Heads DSPE	Thickness [nm]	0.6 ± 0.1	0.6_5 ± 0.1
	SLD [Å^{-2}]	2.6	2.6
	Water [v/v%]	40 ± 5	35 ± 5
	Roughness [nm]	0.5_5 ± 0.2	0.6 ± 0.2
Tails	Thickness [nm]	3.7 ± 0.1	3.9 ± 0.1
	SLD [Å^{-2}]	-0.3	-0.3
	Water [v/v%]	14 ± 5	7 ± 5
	Roughness [nm]	0.5_5 ± 0.2	0.7 ± 0.2

Heads DPPC/CD	thickness [nm]	$0.9_8 \pm 0.1$	$0.9_6 \pm 0.1$
	SLD [$Å^{-2}$]	1.8	1.8
	Water [v/v%]	35 ± 5	25 ± 5
	Roughness [nm]	$0.5_5 \pm 0.2$	$0.8_5 \pm 0.2$
PEG	Thickness [nm]	-	13.0 ± 1.0
	SLD [$Å^{-2}$]	-	0.6
	Volume fraction Φ_0	-	0.08 ± 0.02
	Roughness [nm]	-	0.8 ± 0.2

Supplementary Table 4. Neutron reflectivity results for supported bilayers with a first monolayer DSPE as well as a second mixed monolayer DPPC/cholesteryl β-CD and DPPC/STL, respectively. The obtained results for the polymer layer yield in a STL surface density $\sigma = 0.051$ nm^{-2}, which is in good agreement with the SFA data (see Supplementary Table 2).

Supplementary Methods

1. Synthesis

1.1. Synthesis of the STL

The STL **6** is synthesized in two steps starting from poly(ethylene glycol) bis(amine) **1** (Supplementary Fig. 1). In the first step polyrotaxanes with a controlled, very low threading ratio are formed with azido α-CD (6I-azido-6I-deoxy-cyclomaltohexaose) **2**, threaded onto the PEG chains in water. A small number of CDs per chain is achieved by forming the PEG/CD inclusion complex at high temperatures, which additionally provides sufficient solubility of the poorly soluble modified CD as previously reported [4]. However the reported capping reaction requires the prior preparation of a water soluble blocked isocyanate to yield the blocking urea. Capping reactions in water are not numerous [5]. In order to get a more versatile pathway, we turned to Kunishima's method to form carboxamide using DMT-MM (4-(4,6-Dimethoxy-1,3,5-triazin-2-yl)-4-methylmorpholinium chloride) in protic solvents [6,7]. This was introduced in rotaxane chemistry by Easton & Coll. [8] and more recently used with polyrotaxanes in a mixture of DMSO and water by the group of Yui [9]. The insoluble adamantane carboxylic acid was rendered water soluble as a β-CD complex **3**. It was then reacted in situ with the pseudo-polyrotaxane formed from **1** and **2**. It was observed that the same number of CDs per chain was obtained in the chosen complexation conditions with both capping methods. The adamantane terminated polyrotaxane 4 was thus obtained by simple dialyses in 25% yield.

The final product is obtained by attaching a cholesteryl succinic acid propargylamide **5** to the polyrotaxane **4** via a click chemistry approach, adapting the method recently reported by Finn & Coll. [10] to afford the STL **6**. The cholesteryl succinic acid propargylamide **5** is previously synthesized in one step from propargylamine and cholesteryl hemisuccinate activated as a toluene sulfonic mixed anhydride [11] according to Mukaiyama & Coll. [12]. All products were verified by NMR recorded on a Bruker DMX300 spectrometer and a BB probe. NMR data were processed and plotted using MestRe-C. Extensive dialyses remove salts and soluble small molecules. Filtration removes insoluble matter. NMR signals include only peaks accounting for the polymer and threaded modified α-CD.

1.2. Synthesis of the cholesteryl β-CD receptor

The cholesteryl β-CD **8** is obtained in one step from azido β -CD (7I-azido-7I-deoxy-cyclomaltoheptaose) **7** with the same click chemistry approach already mentioned in section 1.1. using cholesteryl succinic acid propargylamide **5**. Small compounds **5** and **8** show single spot in TLC and no extra peak in NMR.

1.3. Materials

1.3.1 Used Chemicals

The chemicals and solvents used throughout the synthesis are listed in Supplementary Table 1.

1.3.2 Synthesis of the 1-adamantane carboxylic acid/β-CD complex 3

700 mg (0.62 mmol, 1eq) β-CD were dissolved in 50 ml of millipore water, sonicated for 20 min and then heated to 70°C while stirring. Likewise 450 mg (2.5 mmol, 4eq) of 1-adamantane carboxylic acid were dissolved in 50 ml of acetone and added slowly to the β-CD solution via a dropping funnel. Then the transparent mixture was sonicated for 45 min and left stirring at 70°C for 3h to completely evaporate the acetone. 30 ml of water were added to the now turbid mixture. Then it was filtered with a 1 µm fiber glass filter and washed several times with Millipore water. The transparent solution was freeze dried to give **3**.

Yield: 710 mg, (87 %)

^{1}H-NMR (300MHz, DMSO-D6): 5.8 (14H, -OH-1 and -OH-2 CD); 4.8 ppm (7H, H-1 CD); 4.45 ppm (7H, -OH-6 CD); 2.5 ppm (residual H_2O); 1.95 ppm (3H, -C-H adamantane); 1.78 ppm (6H, -CH_2-CCOOH adamantane); 1.65 ppm (6H, -CH_2-CH adamantane)

1.3.3 Synthesis of the polyrotaxane 4

100 mg (10 µmol, 1eq) α,ω-diamino PEG **1** (N = 222, MW = 10000 g/mol) was dissolved in 3.4 ml millipore water and 150 mg (150 µmol, 15eq) α-CDN_3 **2** were added. The transparent solution was left stirring at 70°C for 2h. Then at first 55 mg (40 µmol, 4eq) 1-Adamantanecarboxylic acid/β-CD complex **3** and subsequently 12 mg (40 µmol, 4eq) DMT-MM were added to the solution, which was left stirring at 70°C for 12h. Finally the mixture was diluted with 15 ml of millipore water and dialysed (cut-off 2000 g/mol) four times with 1.5l of millipore water at 50°C. The transparent solution is freeze dried to give **4**.

Yield: 30 mg (25%)

^{1}H-NMR (300MHz, $CDCl_3$): 7.35 ppm (2H, NHCO- stopper); 5.6 ppm - 5.4 ppm (12H, OH-2 and OH-3 CD); 4.8 ppm (6H, H-1 CD); 4.45 ppm (6H, OH-6 CD); 3.1-3.9 (nH, -OCH_2CH_2-PEG and H-2, H-5, CH_2-6 CD); 2.5 ppm (residual H_2O); 1.9 ppm (6H, CH adamantane); 1.7 (24H, CH_2 adamantane)

1.3.4 Synthesis of cholesteryl succinic acid propargylamide 5

2.5 g (5.1 mmol, 1.1 eq) of cholesterol-hemisuccinate, 1.81 g (5.6 mmol, 1.3 eq) of toluenesulfonic anhydride and 1.26 g (10 mmol, 2.2 eq) of DMAP were dissolved in 25 ml of $CHCl_3$ and after 15 min 0.26 g (4.6 mmol, 1 eq) propargylamine were added. After 1.5h the mixture was quenched with 3 ml of saturated $NaHCO_3$ solution. The solution was extracted with ethyl acetate and the combined organic layers were washed two times with 50 ml of saturated $NaHCO_3$, two times with 50 ml of brine and the organic layer was dried over anhydrous Na_2SO_4. The crude product was purified by recrystallization in ethyl acetate and freeze dried from cyclohexane to give **5**.

Yield: 1.25 g (55 %)

^{1}H-NMR (300MHz, $CDCl_3$): 6.2 ppm (1H, -NHCO-); 5.35 ppm (1H, H-6 Cholesterol); 4.6 ppm (1H, H-3 Cholesterol); 4.0 ppm (2H, $-CH_2$ propargyl); 2.2 ppm (1H, H-alkyne); 0.65 ppm (9H, $-CH_3$ cholesterol)

1.3.5 Synthesis of the STL 6

Prior to the experiment solutions of $CuSO_4$ (c = 0.13 mol/l) and a THBTA (c = 63 mmol/l) are prepared with Millipore water. The polyrotaxane **4** (30 mg, 2.8 µmol, 1eq) and the cholesteryl succinic acid propargylamide **5** (6 µmol, 2eq (per azide)) were dissolved in a mixture of 1.5 ml tert-BuOH/Millipore water 8:2, sonicated for 5 min and heated for several minutes to provide for complete dissolution of the compounds. Then the ligand solution (1 µmol, 0.3 eq) and the CuSO4 solution (0.2 µmol, 0.06 eq) were added to the mixture to give a transparent solution. Sodium ascorbate (2.5 µmol, 0.8 eq) was added and the solution was left stirring for 4h at room temperature. The transparent solution was diluted with 5 ml of Millipore water and dialyzed (cut-off 2000 g/mol) twice with 2l of millipore water for 24h and freeze dried. The crude product was taken up in 5 ml of ether and centrifuged 3 times to eliminate the residual cholesteryl succinic acid propargylamide. The residue was dissolved in 10 ml of tert-BuOH/H2O 8:2 and freeze dried to give **6**.

Yield: 13 mg (50%)

^{1}H-NMR (300MHz, $CDCl_3$): 8.3 ppm (1H, NHCO- succinyl); 7.8 ppm (1H, H-triazol); 7.8 ppm (2H, NHCO- stopper); 5.6 ppm - 5.4 ppm (12H, OH-2 and OH-3 CD); 5.3 ppm (1H, CH sp2 cholesterol); 5.0 ppm (1H, H-1 modified glucose unit CD); 4.8 ppm (5H, H-1 CD); 4.5 ppm (6H, OH-6 CD); 3.1-3.9 (nH, $-OCH_2CH_2-$ PEG and H-2, H-5, CH2-6 CD); 2.5 ppm (residual H2O); 1.9 ppm (6H, CH adamantane), 1.7 (24H, CH_2 adamantane); 1.8 - 0.8 ppm (H cholesteryl moiety); 0.65 ppm (9H, $-CH_3$ cholesterol)

1.3.6 Synthesis of the cholesteryl β-CD 8

Prior to the experiment solutions of $CuSO_4$ (c = 0.13 mol/l) and THBTA (c = 63 mmol/l) were prepared with Millipore water. 47 mg (40 µmol, 1eq) β-CDN_3 and 28 mg (56 µmol, 1.4eq) cholesteryl succinic acid propargylamide **5** were introduced into 16 ml of tert-butanol and sonicated for 10 min. Then 228 µl (13 mg, 30 µmol, 0.75 eq) of the THBTA solution and 72 µl (0.77 mg, 5 µmol, 0.1eq) of the $CuSO_4$ solution were mixed in 3.7 ml of water added to the mixture to give a slightly turbid suspension. 40 mg (200 µmol, 5eq) of sodium ascorbate were put into the solution and the mixture was stirred for 1h at room temperature. In the next step the compound was centrifuged three times in 10 ml of buffer/EDTA solution (2 mg EDTA in phosphate buffer pH = 6.5) and three times in 3 ml of acetone. The compound was taken up in 5 ml of Millipore water and freeze dried to give **8** (Supplementary Fig. 2).

Yield: 62 mg (90 %)

^{1}H-NMR (300MHz, DMSO-D6): 8.30, 8.27 ppm (s, 1H, -NHCO-); 7.83, 7,66 ppm (s, 1H, H-triazol); 5.6 ppm - 5.4 ppm (14H, OH-2 and OH-3 CD); 5.0 ppm (1H, H-1 modified glucose unit CD); 4.8 ppm (6H, H-1 CD); 4.4 ppm (7H, OH-6 CD); 3.2-3.7 (H-2 and H-5, CH_2-6 CD); 2.5 ppm (residual H_2O), 2 - 0.8 ppm (H's of cholesteryl moiety); 0.65 ppm (9H, -CH3 cholesterol)

2. Direct Force Measurements (SFA)

2.1. A note on the bare bilayer thicknesses

Note that the added thickness of the two decorated bilayers in Fig. 4 of the main text is slightly larger, by 0.2 nm only, than that of the corresponding bare bilayers. Since the zero reference distance for all force-distance profiles is defined at the contact of the two decorated bilayers as in Fig.1 of main text, some precisions must be added here. First, one can remark that at very short separations the compliance of the steric repulsion is smaller in the presence of STLs compared to the situations where no polymer is present (compare Fig. 4, Fig. 5 and Fig. 6 of the main article). If a contact value can be easily defined for Fig. 6, as the steric repulsion vs. separation appears almost vertical, defining contact values for other cases is not as straightforward. For Fig. 5 this “contact“ (9.6±0.2 nm) corresponds to the thicknesses of the two bilayers when they are brought to contact, while it would be slightly larger by about 0.2 nm for Fig.4 and 0.1-0.2 nm for Fig. 5. For these latter situations the contact value is defined by extrapolation from the slope of the steric repulsion compliance, since we have avoided applying too large loads in order not to damage the structure of the decorated bilayers. In any case, the additional 0.2 nm cannot be interpreted as a thickness layer due to compression of the polymer at infinite loads. It is more likely due to the rearrangement of the ß-CDs in the presence of STLs, which, under high loads, are likely to re-orient and protrude slightly from the bilayer, as seen in [4].

2.2 A note on the role of ligand polydispersity on the attractive forces

We should stress that while compression curves are well explained by a polymer length distribution with average polymerization index N=220 and PDI=1.25 (note that our PDI is well within the bounds provided by Sigma Aldrich that states that PDI < 1.3 for these samples), chains with N~ 600 contribute mostly to the withdrawal forces. This calls for two remarks. First, such an amount of large chains exists indeed in the distribution: for a Flory-Schulz distribution with average length 220 and PDI=1.25, there are ~ 0.34 % of the chains with N>630, comparable to the values in Supplementary Table 3. Moreover, as explained above, our preparation of the STL constructs involves a dialysis step that is likely to skew the original polymer distribution towards the larger chains. Secondly, the predominance of large chains contributing to the attractive forces is likely to be a direct consequence of the conditions for bridging. Indeed, for a given distance at contact between the two opposing surfaces, the probability of bridging increases with the size of the chains.

Supplementary References

(1) Moore, N. W. & Kuhl, T. L. Bimodal polymer mushrooms: Compressive forces and specificity toward receptor surfaces. *Langmuir* **22** (20), 8485-8491 (2006).

(2) Auletta, T., de Jong, M. R., Mulder, A., van Veggel, F. C. J. M., Huskens, J., Reinhoudt, D. N., Zhou, S., Zapotoczny, S., Schönherr, H., Vancso, G. J. & Kuipers, L. ß-Cyclodextrin host-guest complexes probed under thermodynamic equilibrium: Thermodynamics and AFM force spectroscopy. *J. Am. Chem. Soc.* **126** (5), 1577-1584 (2004)

(3) Harries, D., Rau, D. C. & Parsegian, V. A. Solutes probe hydration in specific association of cyclodextrin and adamantane. *J. Am. Chem. Soc.* **127** (7), 2184-2190 (2005).

(4) Bauer, M. Bernhardt, M., Charitat, T., Kékicheff, P., Fajolles, C. Fragneto, G., Marques, C. M. & Daillant, J. Membrane insertion of sliding anchored polymers *Soft Matter* **9**, 1700-1710 (2013).

(5) Fleury, G., Brochon, C., Schlatter, G., Bonnet, G., Lapp, A. & Hadziioannou, G., Synthesis and characterization of high molecular weight polyrotaxanes: towards the control over a wide range of threaded alpha-cyclodextrins. *Soft Matter* **1** (5), 378-385 (2005).

(6) Kunishima, M., Kawachi, C., Morita, J., Terao, K., Iwasaki, F. & Tani, S. 4-(4,6-dimethoxy-1,3,5-triazin-2-yl)-4-methyl-morpholinium chloride: An efficient condensing agent leading to the formation of amides and esters. *Tetrahedron* **55** (46), 13159-13170 (1999).

(7) Kunishima, M., Kawachi, C., Hioki, K., Terao, K. & Tani, S. Formation of carboxamides by direct condensation of carboxylic acids and amines in alcohols using a new alcohol- and water-soluble condensing agent: DMT-MM. *Tetrahedron* **57**, 1551-1558 (2001).

(8) Maniam, S., Cieslinski, M. M., Lincoln, S. F., Onagi, H., Steel, P. J., Willis, A. C. & Easton, C. J. Molecular Fibers and Wires in Solid-State and Solution Self-Assemblies of Cyclodextrin [2]Rotaxanes. *Org. Lett.* **10** (10), 1885-1888 (2008).

(9) Hyun, H. & Yui, N. Mono-, Di-, or Triazidated Cyclodextrin-Based Polyrotaxanes for Facile and Efficient Functionalization via Click Chemistry. *Macromol. Rapid Commun.* **32**, 326-331 (2011).

(10) Hong, V., Presolski, S. I., Ma, C. & Finn, M. G. Analysis and Optimization of Copper-Catalyzed Azide–Alkyne Cycloaddition for Bioconjugation. *Angew. Chem. Int. Ed.* **48** (52), 9879-9883 (2009).

(11) Bauer, M., Fajolles, C., Charitat, T., Wacklin, H. & Daillant, J. Amphiphilic Behavior of New Cholesteryl Cyclodextrins: A Molecular Study. *J. Phys. Chem. B* **115**, 15263-15270 (2011).

(12) Funasaka, S., Kato, K. & Mukaiyama, T. Highly efficient method for the synthesis of carboxamides from carboxylic acids and amines using benzenesulfonic anhydride (BSA). *Chem. Lett.* **36**, 1456-1457 (2007).
